# Effect of Inoculation with *Glomus versiforme* on Cadmium Accumulation, Antioxidant Activities and Phytochelatins of *Solanum photeinocarpum*

**DOI:** 10.1371/journal.pone.0132347

**Published:** 2015-07-15

**Authors:** Shi-Yun Tan, Qiu-Yun Jiang, Feng Zhuo, Hui Liu, Yu-Tao Wang, Shao-Shan Li, Zhi-Hong Ye, Yuan-Xiao Jing

**Affiliations:** 1 Key Laboratory of Ecology and Environmental Science in Guangdong Higher Education, College of Life Sciences, South China Normal University, Guangzhou, P. R. China; 2 State Key Laboratory for Bio-control, and School of Life Sciences, Sun Yat-sen University, Guangzhou, P. R. China; Estación Experimental del Zaidín (CSIC), SPAIN

## Abstract

The plant growth, phosphate acquisition, Cd translocation, phytochelatins (PCs) production and antioxidant parameters [superoxide dismutase (SOD), catalase (CAT), guaiacol peroxidase (POD), ascorbate peroxidase (APX), glutathione reductase (GR), glutathione (GSH), ascorbate (ASA) and malonaldehyde (MDA)] were investigated in Cd-hyperaccumulator *Solanum photeinocarpum* inoculated with *Glomus versiforme* BGC GD01C (Gv) in Cd-added soils (0, 5, 10, 20, 40 mg Cd kg^-1^ soil). Mycorrhizal colonization rates were generally high (from 77% to 94%), and hardly affected by Cd. Gv colonization significantly enhanced P acquisition, growth and total Cd uptakes in both shoots and roots of *S*. *photeinocarpum* at all Cd levels. Meanwhile, Gv symbiosis significantly increased Cd concentration in the roots, and decreased Cd concentration in the shoots at all Cd levels, which indicates that Gv could promote phytostabilization by enhancing Cd accumulation in the roots to inhibit its translocation to shoots and the “dilution effects” linked to an increase in plant dry matter yield and a reduced Cd partitioning to shoots. Moreover, the improvement of CAT, POD and APX activities in the leaves of mycorrhizal plants infers that Gv symbiosis helped *S*. *photeinocarpum* to relieve oxidative damage to biomolecules in Cd-contaminated soil. The evident decline of MDA content in the leaves of mycorrhizal plants indicates that Gv symbiosis evidently improved antioxidant activities, and the enhancement of PCs production in the leaves of mycorrhizal plants suggests that Gv-inoculated plant may be more efficient to relieve Cd phytotoxicity. Therefore, the possible mechanisms of Cd phytotoxicity alleviation by Gv can be concluded as the decline of Cd concentration in the shoots and the improvement of P acquisition, PCs production and activities of CAT, POD, APX in mycorrhizal plants.

## Introduction

Cadmium (Cd) is a non-essential heavy metal (HM) element for plant growth, with high phytotoxicity even at very low level-0.5 mg kg^-1^ soil [1]. Excessive Cd actually causes accumulation of reactive oxygen species (ROS) to damage cellular structure and disturb metabolism, such as lipid peroxidation, electrolyte leakage and leaf photosynthesis inhibition [2]. However, plant cells contain an array of protective and repair systems that minimize the occurrence of oxidative damage. The antioxidative system is composed of antioxidant enzymes, such as superoxide dismutase (SOD), catalase (CAT), ascorbate peroxidase (APX), guaiacol peroxidase (POD), glutathione reductase (GR) etc and some small molecules which regenerate oxidized antioxidants including ascorbate (ASA) and glutathione (GSH) etc [3]. SOD is a major O_2_^-^ scavenger, and its enzymatic action results in H_2_O_2_ and O_2_ formation. CAT, POD and APX have a capacity to convert H_2_O_2_ into H_2_O [4]. GR and GSH play an important role in removing H_2_O_2_ by the ascorbate-glutathione pathway [5]. Excessive ROS can lead to membrane lipid peroxidation, which can be assessed by measuring the concentration of malonaldehyde (MDA) [6]. Phytochelatins (PCs) form complexes with metals through their cysteine sulfhydryl groups and these metal complexes are subsequently compartmentalized in the vacuole, which has been considered as an important detoxification mechanism for toxic metals in plants [7]. PCs synthesis in cells is directly related to metal stress in plants, and therefore analysis of PCs could be a useful biomarker for the estimation of metal toxicity [8].

With the rapid development of industry, Cd-contamination in the soils has become a severe environmental problem throughout the world. To remedy Cd-contaminated soils, a range of environmental remediation methods using physical, chemical or biological strategies have been developed [9–10]. Of these, phytoremediation, utilizing plants to remove, transfer, stabilize, decrease, and decompose pollutants in the environment, is a low cost and environmentally friendly technology [11]. However, successful phytoremediation is not easy to achieve due to phytotoxicity at high HM concentrations and small biomass production by the applied plants [12]. The roles of arbuscular mycorrhizal **(**AM) fungi in HM stress alleviation of plants exposed to HM-contaminated soils and their contribution to the phytoremediation process have already been recognized [11]. AM fungi can improve nutrition acquisition of plant [13–15] and provide a HM isolation belt to protect plant against HM toxicity [16–17]. Meanwhile, AM fungi can also prevent plant from absorbing HM by secreting some organic compounds (such as glomalin) in soil to chelate metal ions [18]. Hildebrandt et al [19] found several genes in AM fungi with putative roles in relieving oxidative stress, therefore, a major function of AM fungi could be to protect plants against HM-induced oxidative stress. However, the overall mechanisms by which AM fungi relieves HM toxicity in host plants are still not completely elucidated, with disputable results depending on the interactions of specific plant, fungal and metal species.

Most metal hyperaccumulator plants, including those in families such as Brassicaceae, Plumbaginaceae, Juncaceae, Caryophyllaceae, Juncaginaceae and Amaranthaceae do not form arbuscular mycorrhizas [11], although the AM symbiosis has been found in some hyperaccumulator plants in recent years [20–22]. *Solanum photeinocarpum* Nakamura et Odashima is found to be a Cd-hyperaccumulator, and has a strong potential to remedy Cd-contaminated soils [23]. However, to our knowledge, there has been no report to elucidate the role of AM fungi in Cd accumulation and Cd phytotoxicity alleviation in this plant grown in Cd-contaminated soil. In this study, we explored the effect of *G*. *versiforme* (Karsten) Berch BGC GD01C (Gv) on plant growth, Cd accumulation, antioxidant system and PCs production in *S*. *photeinocarpum* in different Cd-added soils (0, 5, 10, 20 and 40 mg Cd kg^-1^ soil), which will provide new insights into the mechanism of Cd phytotoxicity alleviation by Gv in mycorrhizal plant.

## Materials and Methods

### Soil preparation

Sampling of the experiment soil was as described by He et al. [24], and the basic properties of the soil were as follows: pH 6.85 (1:1 w/v water), organic matter 1.65%, total N 1.74 g kg^-1^, total P 0.55 g kg^-1^, total K 14.35 g kg^-1^, available P 52 mg kg^-1^, total Cd 0.14 mg kg^-1^ and diethylenetriamine pentaacetic acid (DTPA)-extractable Cd 0.063 mg kg^-1^. The soil was autoclave-sterilized (121°C for 1.5 h) after sieved (2 mm), then divided into five aliquots amended with Cd concentrations of 0 (control), 5, 10, 20, 40 mg Cd kg^-1^ soil (supplied as CdCl_2_), respectively. Before use, the Cd-amended soils were subjected to saturation with sterile water for two months to stabilize metal.

### Preparation of AM fungus

The *G*. *versiforme* BGC GD01C (Gv) obtained from the Beijing Academy of Agriculture and Forestry, China, was isolated from *Pteris vittata* grown in an ancient Pb/Zn mine at Shaoguan, Guangdong Province, China. Gv was propagated on *Zea mays* grown in 2-L pots filled with autoclaved sand and soil (1:1, v/v). Four months later, the roots harvested were cut into pieces and evenly mixed with the culture medium containing spores and hyphae, all of the mixtures were prepared as mycorrhizal inocula.

### Experimental design and pot experiment

The pot experiment was a 5×2 complete factorial combination, which is comprised of five Cd treatments (0, 5, 10, 20 and 40 mg Cd kg^-1^soil) and two mycorrhizal inoculations (with/without Gv). Each treatment was replicated five times in a randomized block design.

The soils (1.5 kg) mentioned above were transferred into each plastic pot (top diameter 22 cm, bottom 17 cm and height 18 cm). Inoculation treatment was conducted by mixing 60 g freshly reproduced Gv inocula in each pot. The sterilized inocula (autoclaved at 121°C for 2 h) were used as a non-inoculation treatment. Each pot of the uninoculated treatment was added with 15 ml filtrate obtained from the unsterilized soil solution sieved through an 11 μm sieve to balance the partial loss of soil bacteria by sterilization [21]. The seeds of *S*. *photeinocarpum* were surface-sterilized in 10% NaClO for 10 min, and then washed with sterile water. The seeds were then placed in a Petri dish filled with sterilized vermiculite, and were allowed to germinate at 25°C and a 16/8 day/night regime. After germination (5 days), six uniform seedlings of *S*. *photeinocarpum* were selected to transplant into each pot. The plants were grown in a controlled greenhouse at temperature 22~28°C with 14/10 day/night regime and the soil was moistened with sterile water and maintained at 60% of its holding capacity. One week later, the seedlings in each pot were thinned to four plants.

### Sampling

After 10 weeks, all plants from each pot were harvested, and divided into shoots and roots. The weighed subsamples of fresh leaves were lyophilized and kept stored in vacuum desiccators for the determination of the PCs production and antioxidant parameters. Roots were immersed in 0.01 M ethylene diamine tetraacetic acid (EDTA) for 30 min, and rinsed subsequently with deionized water to remove metals adsorbed on root surface [25]. Meanwhile, soils in the rhizosphere of the plants were collected to prepare for biochemistry analysis.

### Plant analysis

Subsamples of shoots and roots were dried at 80°C for 3 days to constant weight for determining dry weight, and the dried tissues were used to analyze Cd and P concentration after grinded and digested in a tri-acid mixture (5:1:1 HNO_3_:H_2_SO_4_:HClO_4_) at 225°C by atomic absorption spectrophotometer (AAS) (Z-2000, Hitachi, Japan) and molybdenum–ascorbic acid spectrophotometer (UV-1061, Shimadzu, Kyoto) [26], respectively.

Mycorrhizal colonization was estimated using the methods described by Phillips and Hayman [27] with a slight modification. The fine roots were cut into fragments about 1 cm and dipped in 10% KOH solution, and then placed in 10% H_2_O_2_ (30 min) for blanching and 1% HCl (3 min) for neutralization respectively. Subsequently, the roots were stained by glycerol-trypan blue solution (0.05%) in 90°C for 30 min and dipped in lactic acid-glycerol solution (v/v 1:1). Forty pieces of root samples at each pot were assessed and the rates of mycorrhizal colonization were calculated using the grid-line intersect method [28].

SOD activity was assayed on the basis of SOD’s ability to inhibit the reduction of nitroblue tetrazolium (NBT) by superoxide radicals generated photochemically [29]. One unit of SOD was defined as the amount of enzyme required to inhibit the reduction rate of NBT by 50%. CAT activity was assayed by determining the consumption rate of H_2_O_2_ at 240 nm [30]. The POD activity was assayed by following the change of absorption at 470 nm due to guaiacol oxidation [31]. APX activity analysis was based on APX’s ability to catalyze the oxidation of ascorbate with the presence of H_2_O_2_, and the decrease of absorbance in 1 min was recorded at 290 nm [32]. GR activity was estimated using NADPH, and measured the decrease of absorbance at 340 nm as NADPH oxidation [33]. The ASA was determined as Law et al. [34] described method using dipyridyl as the substrate. MDA was assessed by thiobarbituric acid (TBA) reaction according the method of Heath and Packer [35].

GSH content was determined by o-phthaldialdehyde (OPA)-fluorometry [36]. Plant sample was homogenized in an ice bath with phosphate buffer (pH 8.0) containing 5 mM EDTA and 25% metaphosphoric acid, then centrifuged at 10000 rpm for 20 min at 4°C, the supernatant was incubated with OPA for 15 min before fluorescence detection, and GSH content was measured fluorometrically at 420 nm after excitation at 350 nm. PCs production was evaluated as the D-value between non-protein thiols (NPT) and GSH [37]. The NPT was estimated as described by Ellman [38]. Plant sample was homogenized with 5% sulfosalicylic acid in an ice bath and centrifuged at 10000 rpm for 20 min at 4°C. The supernatant incubated with 5’-dithiobis-2-nitrobenzoic acid (DTNB) for 20 min at 30°C, and the absorbance at 412 nm was measured.

### Soil analysis

Soil DTPA-extractable Cd concentrations were determined by AAS after extraction for 2 h in leach liquor [0.005M DTPA, 0.1 M trie-thanolamine (TEA) and 0.01 M CaCl_2_, pH 7.3, solution:soil = 2:1] [39].

### Statistic analysis

All results were tested by one- or two-way analysis of variance (ANOVA) using the SPSS statistical package. To detect the statistical significance of differences (*p* < 0.05) between different Cd concentrations for all parameters, the Least Significant Difference (LSD) test was performed.

## Results

The results of two-way ANOVA with AM inoculation, Cd concentration and their interaction are given in Table 1.

**Table 1:** Significance level of effects of different factors and factor interactions on variables based on two-way analyses of variance (ANOVA).

Variables	*F*-values		
	AM inoculation	Cd concentration	Cd * AM
Shoot biomass	493.53 ***	20.740 ***	7.78 ***
Root biomass	120.26 ***	14.146 ***	5.02 **
Shoot P concentration	225.99 ***	1.64 NS	4.409 **
Root P concentration	70.79 ***	22.85 ***	3.14 *
Shoot Cd concentration	36.25 ***	334.94 ***	8.841 ***
Root Cd concentration	142.41 ***	131.678 ***	14.98 ***
Shoot Cd uptake	178.22 ***	329.06 ***	11.62 ***
Root Cd uptake	339.21 ***	135.33 ***	12.92 ***
DTPA-extractable Cd concentration	0.062 NS	326.53 ***	0.533 NS
CAT activity	98.76 ***	49.41 ***	2.56 NS
POD activity	106.83 ***	4.03 *	13.95 ***
APX activity	44.01 ***	12.04 ***	5.65 **
SOD activity	0.44 NS	0.91 NS	1.26 NS
GR activity	0.03 NS	6.51 **	4.49 **
GSH content	1.33 NS	2.15 NS	0.34 NS
ASA content	0.002 NS	16.82 ***	1.39 NS
MDA content	299.18 ***	14.69 ***	3.71 *
PCs content	70.61 ***	65.55 ***	9.35 ***

CAT, POD, APX, SOD, GR, GSH, ASA, MDA and PCs represent catalase, guaiacol peroxidase, ascorbate peroxidase, superoxide dismutase, glutathione reductase, reduced glutathione, ascorbate, malonaldehyde and phytochelatins, respectively. NS–not significant;

*–significant at the level *p* < 0.05

**–significant at the level *p* < 0.01

***–significant at the level *p* < 0.001.

### Mycorrhizal colonization

**Fig 1:**
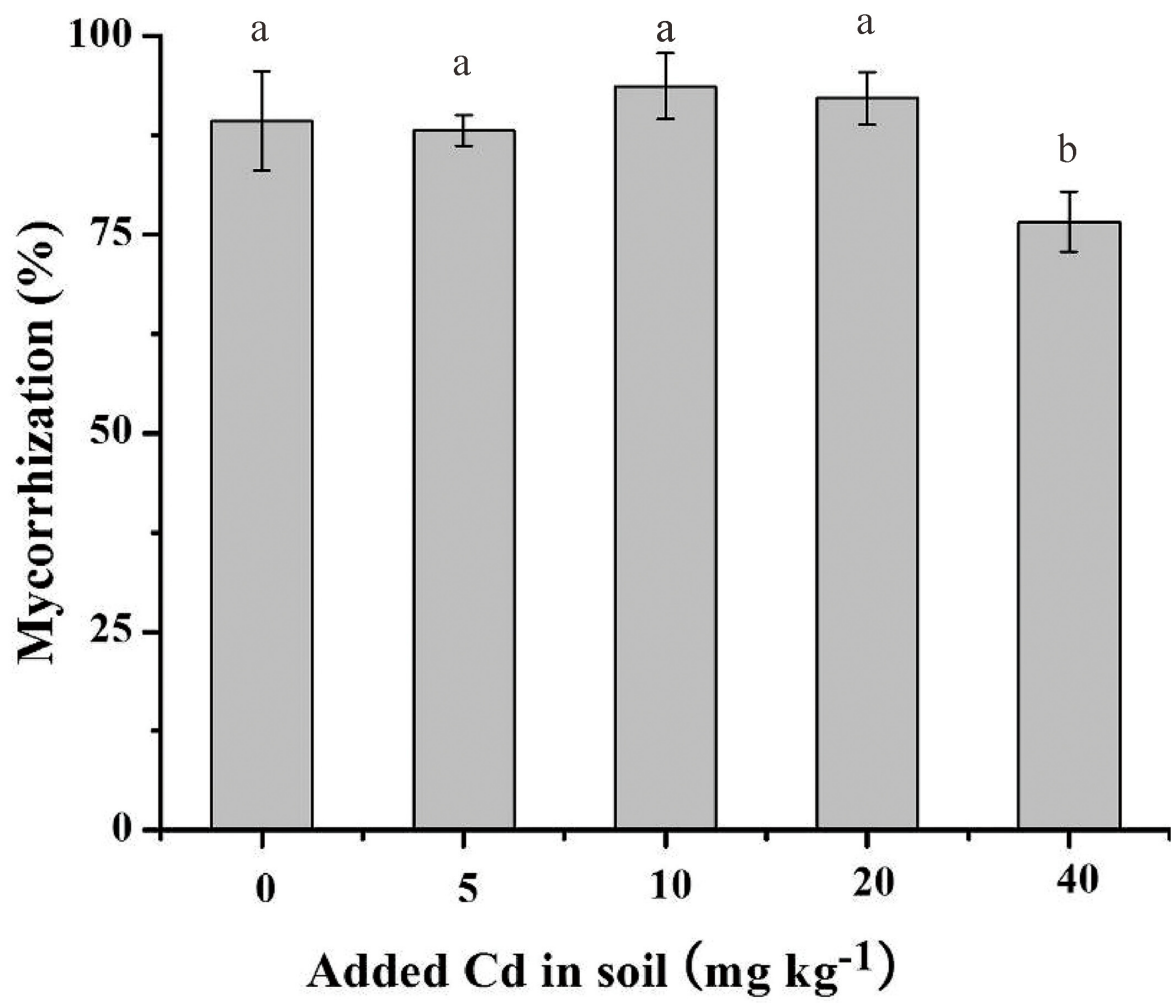
Colonization rates of *S*. *photeinocarpum*. Values are presented as means ± SD for the five replicates. Different letters indicate a significant difference (*p* < 0.05) according to the LSD test.

Gv colonization in the roots of *S*. *photeinocarpum* was founded in the all inoculated treatments (Fig 1), and the colonization rates were generally high (from 77% to 94%). Compared with the Cd-unamended soil, colonization rates were hardly affected in 5, 10 and 20 mg Cd kg^-1^ soils, but was significantly (*p* < 0.05) decreased in 40 mg Cd kg^-1^ soils. Moreover, no hyphae or vesicles were observed in the uninoculated treatments.

### Plant growth and P concentration

**Fig 2:**
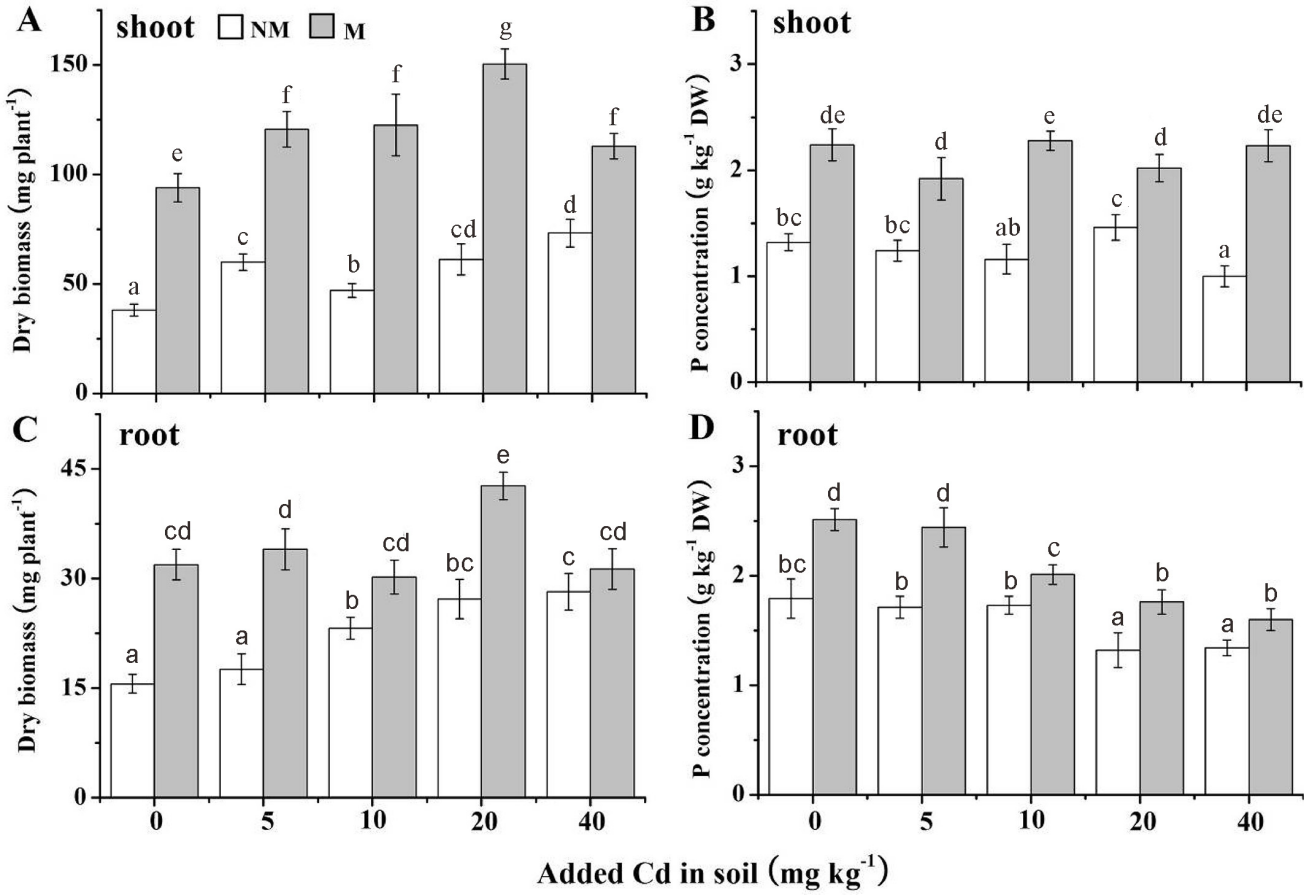
Dry biomasses and P concentrations of *S*. *photeinocarpum*. M **=** mycorrhizal inoculum; NM = non-mycorrhizal inoculum. Values are presented as means ± SD for the five replicates. Different letters indicate a significant difference (*p* < 0.05) according to the LSD test.

The dry biomasses and P concentrations of roots and shoots of *S*. *photeinocarpum* with/without Gv in the different Cd treatments were shown in Fig 2. Except the root biomass in 40 mg Cd kg^-1^ soils, there were significant increases (*p* < 0.05) in biomass of *S*. *photeinocarpum* inoculated with Gv in the all Cd treatments compared with the uninoculated controls, which were from 54% to 161% in the shoots (Fig 2A) and 11% to 105% in the roots (Fig 2C), respectively. Similarly, the Gv colonization significantly (*p* < 0.05) increased P content of *S*. *photeinocarpum* compared with the non-inoculated controls, with the increases from 38% to 122% in the shoots (Fig 2B) and from 16% to 42% in the roots (Fig 2D), respectively.

### Cd accumulation by *S*. *photeinocarpum* and soil DTPA-extractable Cd

**Fig 3:**
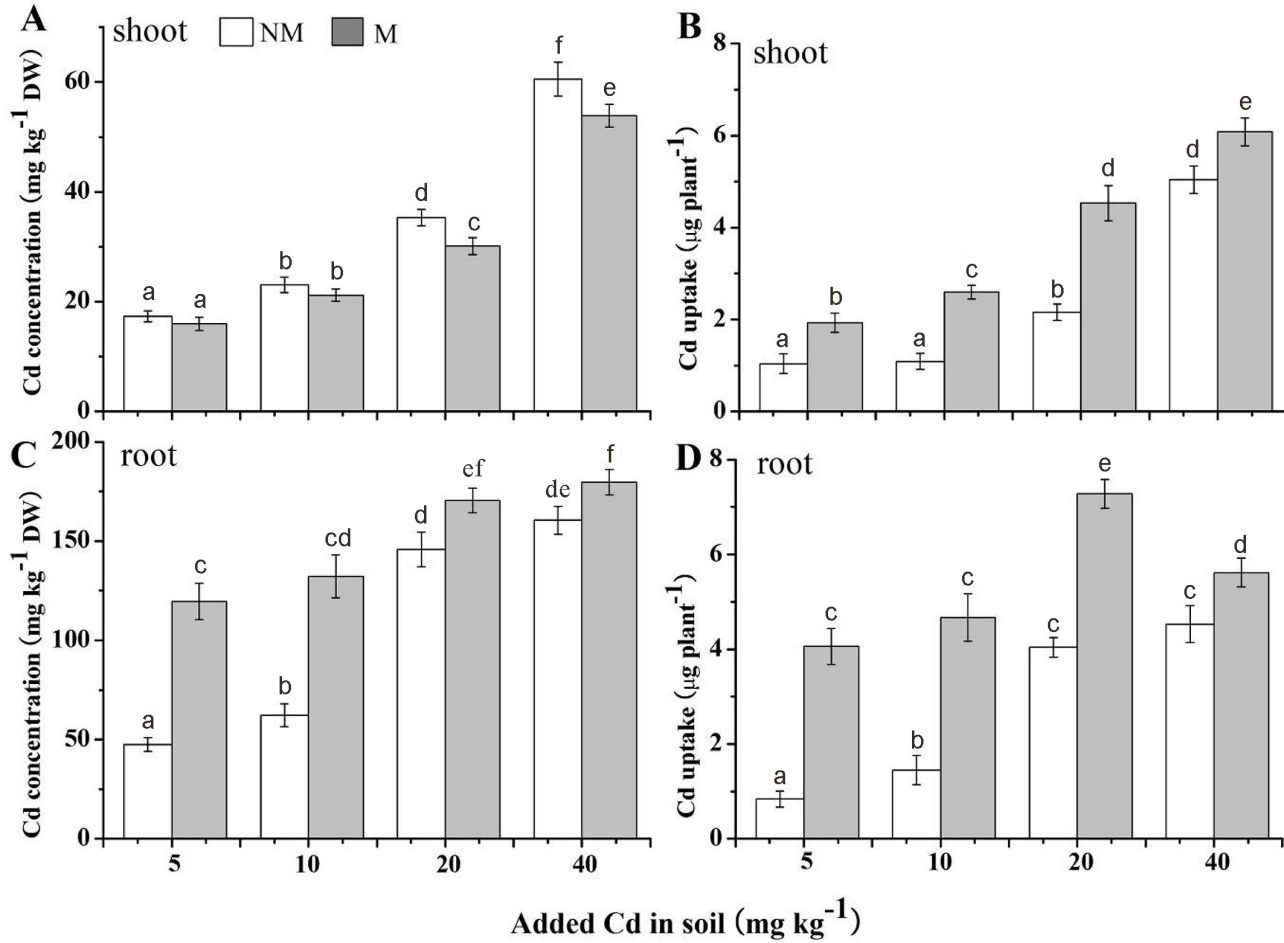
Cd concentrations and Cd uptakes in *S*. *photeinocarpum*. M **=** mycorrhizal inoculum; NM = non-mycorrhizal inoculum. Values are presented as means ± SD for the five replicates. Different letters indicate a significant difference (*p* < 0.05) according to the LSD test.

**Fig 4:**
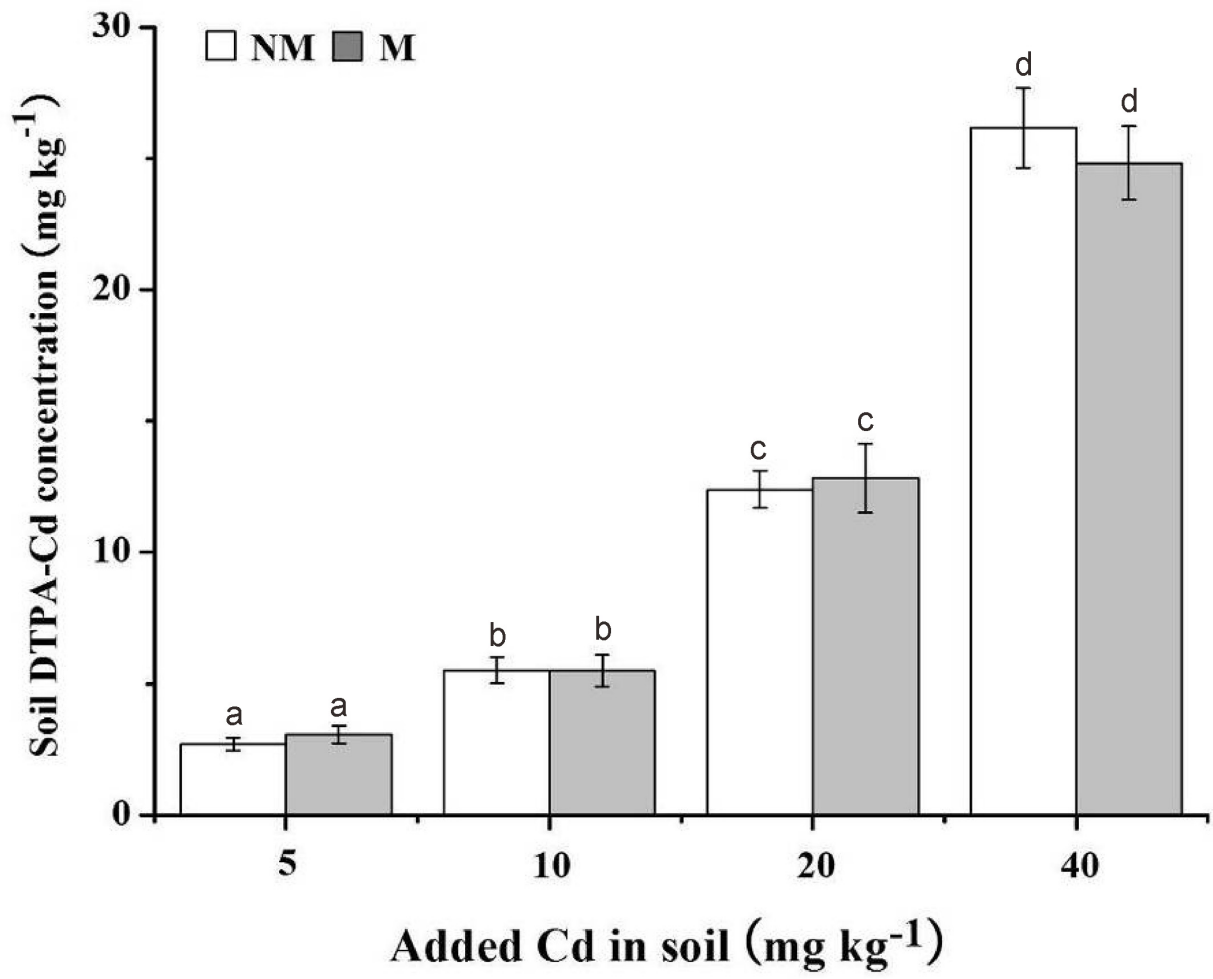
Soil DTPA-extractable Cd concentrations. M **=** mycorrhizal inoculum; NM = non-mycorrhizal inoculum. Values are presented as means ± SD for the five replicates. Different letters indicate a significant difference (*p* < 0.05) according to the LSD test.

The Cd concentrations and Cd uptakes in *S*. *photeinocarpum* with/without Gv in different Cd treatments were showed in Fig 3. Gv colonization decreased Cd concentrations in the shoots, with the decreases from 8% to 15% (Fig 3A), but significantly (*p <* 0.05) increased Cd concentrations in the roots, with the increases from 12% to 151% (Fig 3C), compared with the non-inoculated controls. Moreover, Cd uptakes by mycorrhizal plants were significantly enhanced (*p <* 0.05) at the all Cd levels compared with the uninoculated controls, with the increases from 21% to 139% in the shoots (Fig 3B), and from 24% to 386% in the roots (Fig 3D), respectively. In addition, Gv colonization exerted no influence on soil DTPA-extractable Cd concentrations (Fig 4).

### Antioxidative parameters

**Fig 5:**
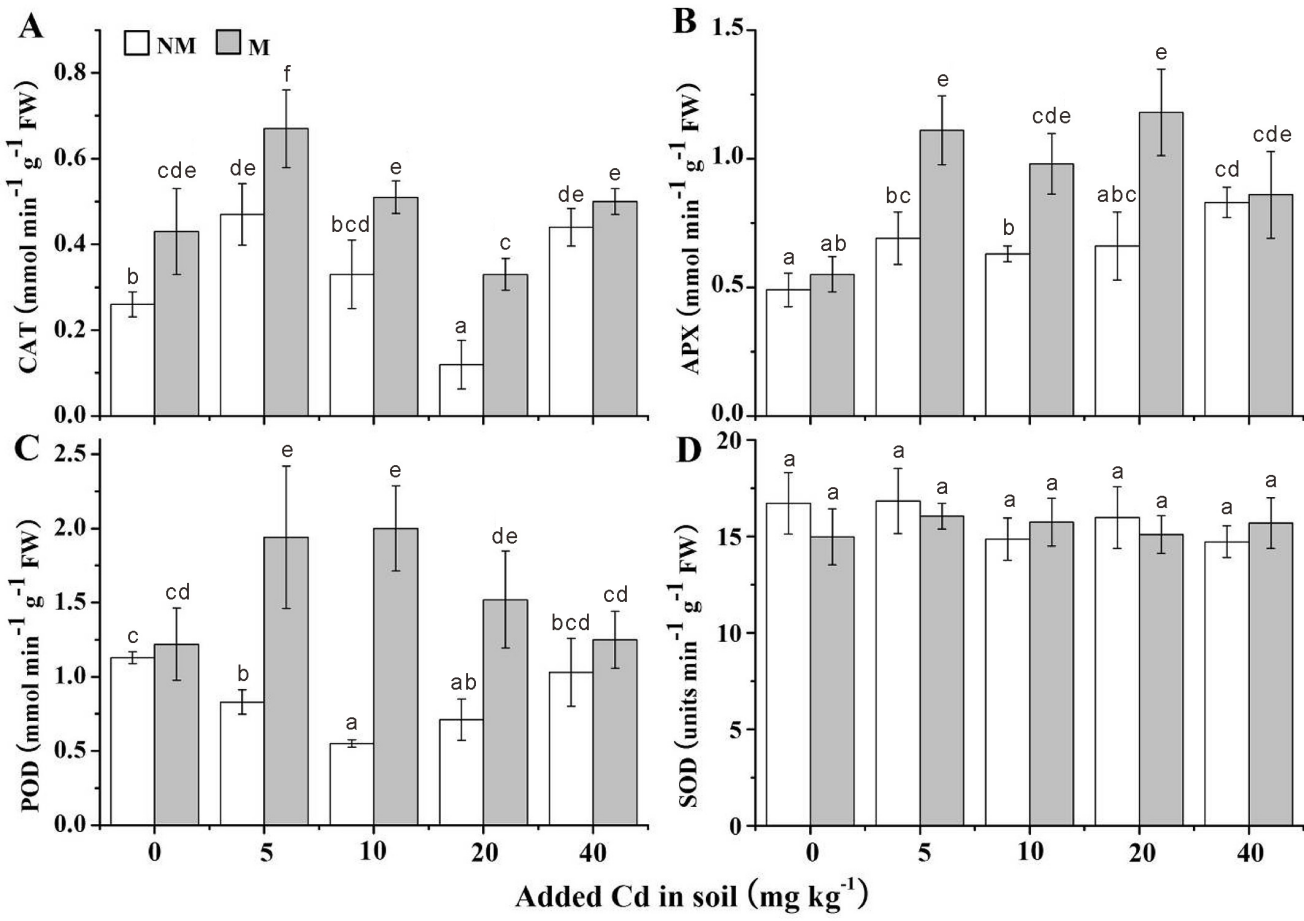
CAT, APX, POD and SOD activities in the leaves of *S*. *photeinocarpum*. CAT, APX, POD and SOD represent catalase, ascorbate peroxidase, guaiacol peroxidase and superoxide dismutase, respectively. M **=** mycorrhizal inoculum; NM = non-mycorrhizal inoculum. Values are presented as means ± SD for the five replicates. Different letters indicate a significant difference (*p* < 0.05) according to the LSD test.

**Fig 6:**
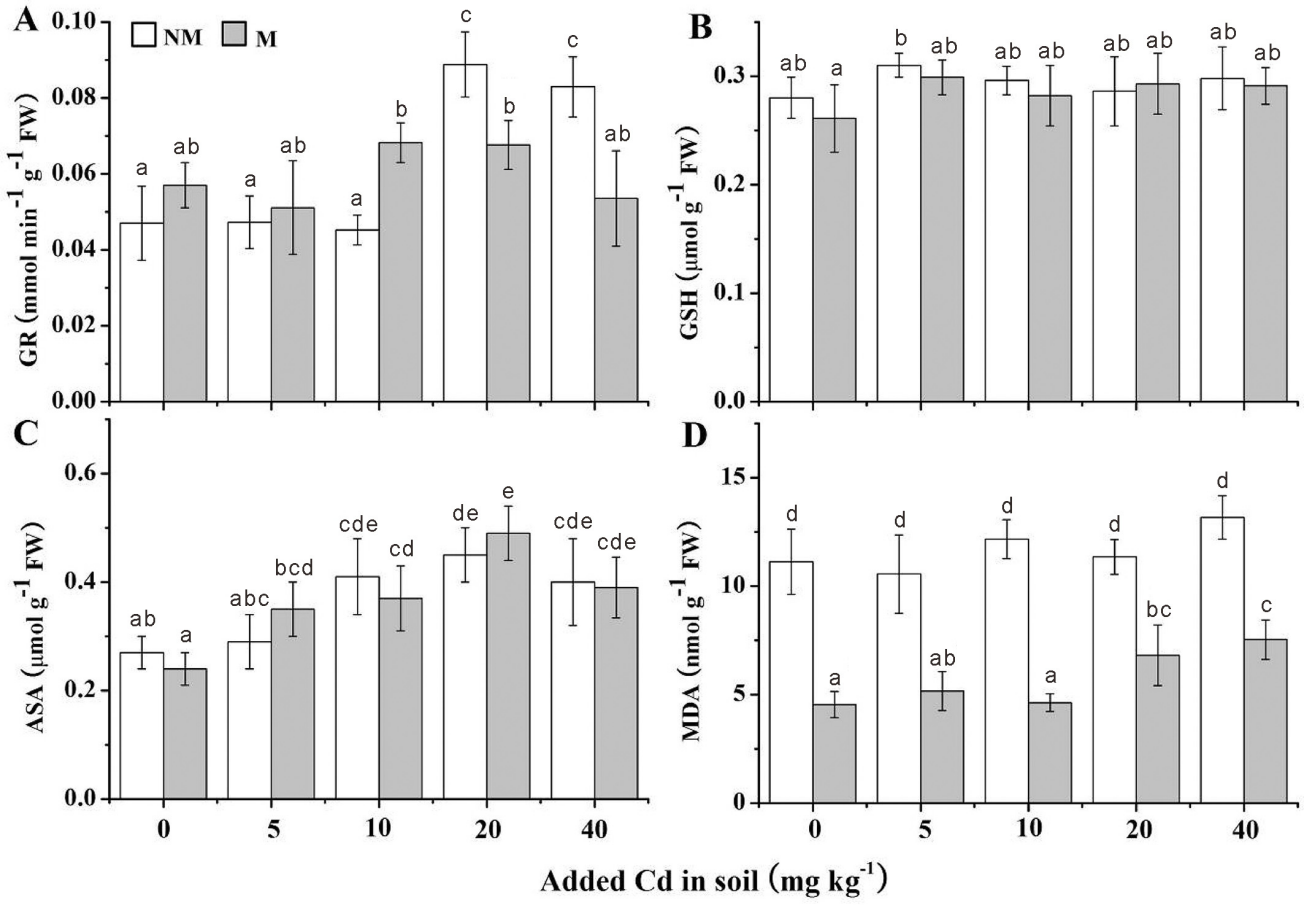
GR activity, and contents of GSH, ASA and MDA in the leaves of *S*. *photeinocarpum*. GR, GSH, ASA and MDA represent glutathione reductase, reduced glutathione, ascorbate and malonaldehyde, respectively. M **=** mycorrhizal inoculum; NM = non-mycorrhizal inoculum. Values are presented as means ± SD for the five replicates. Different letters indicate a significant difference (*p* < 0.05) according to the LSD test.

All the antioxidative parameters of *S*. *photeinocarpum* with/without Gv were showed in Figs 5 and 6. Compared with the non-inoculated controls, Gv symbiosis enhanced the activities of CAT, APX and POD in the all Cd levels, with the increases from 14% to 166% in CAT (Fig 5A), 3% to 79% in APX (Fig 5B) and 8% to 266% in POD (Fig 5C), respectively. However, the Gv symbiosis exerted no influence on SOD activities (Fig 5D). Moreover, the GR activities of mycorrhizal plants were increased in the 0, 5 and 10 mg Cd kg^-1^ soils, but significantly (*p <* 0.05) decreased in 20 and 40 mg Cd kg^-1^ soils compared with non-mycorrhizal plants, respectively (Fig 6A).

Acting as antioxidants, GSH and ASA contents were almost responseless to Gv colonization, and the GSH contents maintained in a stable level in mycorrhizal and non-mycorrhizal plants at all Cd levels (Fig 6B and 6C). In addition, Gv colonization significantly (*p* < 0.05) decreased MDA contents at the all Cd levels, with the decreases from 40% to 62% compared with non-Gv controls (Fig 6D).

### Phytochelatins

**Fig 7:**
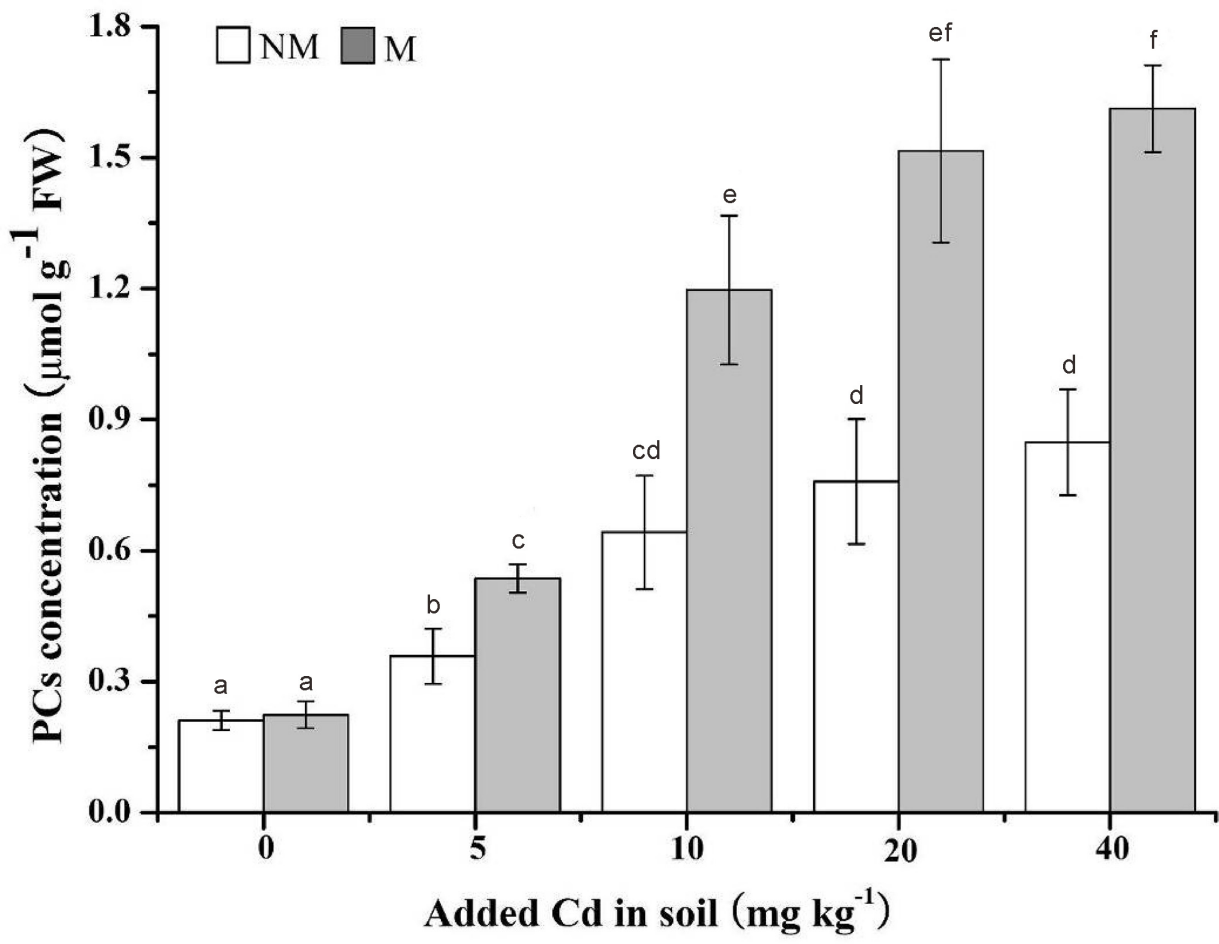
Phytochelatins (PCs) production in the leaves of *S*. *photeinocarpum*. M **=** mycorrhizal inoculum; NM = non-mycorrhizal inoculum. Values are presented as means ± SD for the five replicates. Different letters indicate a significant difference (*p* < 0.05) according to the LSD test.

The production of PCs in mycorrhizal plants was significantly (*p* < 0.05) improved in all Cd levels except the Cd-unamended soil, with the increases from 50% to 100%, compared with non-mycorrhizal plants (Fig 7). Furthermore, the PCs contents in mycorrhizal and non-mycorrhizal plants were increased with the increase of soil Cd concentrations (Fig 7).

## Discussion

AM fungi collected from HM-contaminated sites are considered to be well adapted to HMs, but mycorrhizal colonization in metal-tolerant plant species will be greatly inhibited when HM concentrations in the soil exceed certain thresholds [11]. For example, the addition of As does not inhibit AM fungi colonization rates in upland rice at a low As level (40 mg kg^-1^ soil), but AM fungi colonization is greatly reduced at high As concentration (80 mg kg^-1^ soil) [40]. Similar results were also observed in our experiments. The colonization rates of Gv to *S*. *photeinocarpum* were relatively high (more than 77%) in all inoculated treatments, which indicates that Gv has a very high degree of tolerance to Cd and can associate with *S*. *photeinocarpum* very well.

It is known that AM fungus inoculation has an effect to promote plant growth under HM-contaminated conditions [3, 20, 41], which might be benefit from AM fungi with the functions to balance the micronutrients and enhance nutrient supply especially P [42–43], even promote the soil enzymes activity such as acid phosphatase [25]. In our study, the biomass and P nutrient acquisition were significantly improved in roots and shoots of *S*. *photeinocarpum* with Gv inoculation. Our outcomes are consistent with some previous findings [40, 44, 45], in which AM fungi colonization improved soil acid phosphatase activity, and thus elevated P acquisition and plant growth. The fact that Gv symbiosis evidently increased biomass of *S*. *photeinocarpum* in spite of the presence of considerable amounts of Cd indicates the positive influence of plant–microbe interaction on plant growth.

In spite of the recognition of AM fungi ability to modify HM uptake by plants, controversial reports exist for both enhancement and decrease of plant HM contents due to mycorrhization. Some results have indicated that AM fungi could promote HM uptake and translocation to shoots, and hence significantly improve the phytoextraction [46–48]. However, most studies have shown that AM fungi could promote phytostabilization by enhancing HM accumulation in plant roots to inhibit HM translocation to above-ground tissues. For example, the inoculation with *Funneliformis geosporum* enhances root Cu concentrations, as compared with uninoculated plants, but decreases shoot Cu concentrations in *Aster tripolium* grown in 2 mM Cu kg^-1^ soils [49]. Moreover, Bissonnette et al. [50] also found that the colonization of *Rhizophagus irregularis* increases Cd concentrations in the roots, but decreases Cd concentrations in the shoots in *Salix viminalis*. Similar results were also found in our experiments. The reasons for the decreased shoot Cd concentrations in mycorrhizal *S*. *photeinocarpum* might be (1) that the hyphae in mycorrhiza act as a Cd pool with capacity of adsorbing and binding Cd by different immobilization mechanisms to inhibit Cd translocation to above-ground tissues [11,42], and (2) the “dilution effects” linked to an increase in plant dry matter yield and a reduced Cd partitioning to shoots [51]. In addition, AM fungi can increase HM uptake/concentration of HM-accumulating plants, and thereby decrease HM accumulation by neighboring edible crops, which provides clues for simultaneous efficient remediation of HM-contamination and crop safe production from HM-contaminated soil [52–53].

A number of studies have indicated that AM symbiosis can increase the antioxidant activities to relieve oxidative damage and enhance HM tolerance of plants under HM stress. For example, Chen et al. [54] found that *F*. *mosseae* inoculation increases activities of SOD and CAT in *Populus euphratica* grown in Pb-contaminated soil. Similarly, Rozpadek et al. [55] also found that the presence of *Rhizophagus irregularis* increases activities of SOD, CAT and POD in *Cichorium intybus* in the Cd/Pb/Zn-contaminated soils. Furthermore, Garg and Aggarwal [56] also reported that *F*. *mosseae* colonization significantly improves activities of SOD, CAT, POD, GR and GSH contents in *Cajanus cajan* in the Cd and/or Pb contaminated soils. CAT, POD and APX are involved in the dismutation of hydrogen peroxide to water and molecular oxygen, and they have a synergistic effect on cellular H_2_O_2_ scavenging. In this work, the improvement of CAT, POD and APX activities in mycorrhizal plants suggests that Gv symbiosis helped *S*. *photeinocarpum* to relieve oxidative damage to biomolecules in Cd-contaminated soil. On the other hand, the contents of ASA and GSH in *S*. *photeinocarpum* were hardly influenced by Gv inoculation, which indicates Gv colonization exerted no impact on the ascorbate-glutathione pathway.

Under Cd stress, lipid peroxidation is initiated as a result of oxidative stress. The high accumulation of MDA indicates severe lipid peroxidation. In the present study, the significant decline of MDA content in mycorrhizal plants indicates that the Gv symbiosis evidently reduced ROS level in *S*. *photeinocarpum*, and resulted in oxidantive stress weakness. Similar results were also reported by Abdel Latef [57] that *Capsicum annuum* inoculated with *F*. *mosseae* shows lower MDA contents than uninoculated plants under Cu stress.

There have been a number of evidences indicated that HM can induce the production of PCs in different plant species [58–60]. However, the information about the effect of AM fungi inoculation on PCs synthesis is scanty, and the conclusions are discrepant in different reports. For example, *F*. *mosseae* infection evidently increases PCs contents in roots of *Cajanus cajan* under Cd and/or Zn stress [61–62]. However, Andrade et al. [43] reported that PCs contents in leaves of *Canavalia ensiformis* with *G*. *etunicatum* are reduced in lower Cu levels (0 and 15 mg Cu kg^-1^ soils), but enhanced in higher Cu levels (150 and 450 mg Cu kg^-1^ soils), compared with the non-mycorrhizal plants. In the present study, the improvement of PCs production in mycorrhizal plants infers that Gv-inoculated plants may be more efficient to relieve Cd phytotoxicity.

The hormesis, a concentration-response phenomenon characterized by low-dose stimulation and high-dose inhibition [63] has been found in Cd-hyperaccumulators. For example, biomasses of Cd-hyperaccumulator *Amaranthus hypochondriacus* and *Amaranthus mangostanu* are increased at lower Cd concentrations (25 and 50 mg kg^-1^), but decreased at higher Cd concentration (100 mg kg^-1^) [64]. Similarly, low Cd concentrations (≤2.5mg L^-1^) in solution significantly increase the biomass of Cd-hyperaccumulator *Lonicera japonica*, but high Cd concentrations (≥5 mg L^-1^) significantly reduces biomass [1]. Also growth stimulation at lower Cd concentrations (≤30 mg kg^-1^) and growth inhibition at higher Cd concentration (60 and 100 mg kg^-1^) are observed in *S*. *photeinocarpum* [23]. Hormesis holds true in our experiments, both root and shoot biomasses of nonmycorrhizal or mycorrhizal plants grown in 5, 10, 20 and 40 mg Cd kg^-1^ soil were increased, compared with the plants grown in the Cd-unamended soil.

## Conclusions

In the present study, the effects of Gv inoculation on Cd accumulation and several physiological indices have been investigated in *S*. *photeinocarpum* grown in Cd-added soils, and conclusions are made as follows: Firstly, the Gv could associate with *S*. *photeinocarpum* very well, and then significantly promoted P acquisition, growth and total Cd uptakes in both roots and shoots of *S*. *photeinocarpum*. Secondly, the presence of Gv significantly increased Cd concentrations in the roots, but decreased Cd concentrations in the shoots of *S*. *photeinocarpum*. Finally, CAT, POD and APX activities and PCs production in mycorrhizal plant were higher than those of non-mycorrhizal plant, but the lower MDA contents were detected in the inoculated treatments. In order to further clarify the mechanisms of Cd toxicity alleviation by AM fungi, we will devote to the molecular biology and proteomics researches in *S*. *photeinocarpum* by AM symbiosis grown in Cd-contaminated soil.
